# Baseline relative eosinophil count as a predictive biomarker for ipilimumab treatment in advanced melanoma

**DOI:** 10.18632/oncotarget.19748

**Published:** 2017-08-01

**Authors:** Pier Francesco Ferrucci, Sara Gandini, Emilia Cocorocchio, Laura Pala, Federica Baldini, Massimo Mosconi, Gian Carlo Antonini Cappellini, Elena Albertazzi, Chiara Martinoli

**Affiliations:** ^1^ Medical Oncology of Melanoma Unit, Division of Medical Oncology of Melanoma and Sarcoma, European Institute of Oncology, Milan, Italy; ^2^ Division of Epidemiology and Biostatistics, European Institute of Oncology, Milan, Italy; ^3^ Division of Surgery of Melanoma and Sarcoma, European Institute of Oncology, Milan, Italy; ^4^ IV Oncology Division, Istituto Dermopatico dell’Immacolata IRCCS, Rome, Italy

**Keywords:** eosinophil, predictive, biomarker, ipilimumab, melanoma

## Abstract

As diverse therapeutic options are now available for advanced melanoma patients, predictive markers that may assist treatment decision are needed. A model based on baseline serum lactate dehydrogenase (LDH), peripheral blood relative lymphocyte counts (RLC) and eosinophil counts (REC) and pattern of distant metastasis, has been recently proposed for pembrolizumab-treated patients. Here, we applied this model to advanced melanoma patients receiving chemotherapy (*n =* 116) or anti-CTLA-4 therapy (*n =* 128). Visceral involvement, LDH and RLC were associated with prognosis regardless of treatment. Instead, when compared to chemotherapy-treated patients with REC < 1.5%, those with REC ≥ 1.5% had improved overall survival when receiving anti-CTLA-4 [Hazard Ratio (HR) = 0.56 (0.4–0.93)] but not chemotherapy [HR = 1.13, (0.74–1.74)], and the treatment-by-REC interaction was significant for both overall (*p* = 0.04) and progression free survival (*p* = 0.009). These results indicate baseline REC ≥ 1.5% as a candidate predictive biomarker for benefit from anti-CTLA-4. Further studies are needed to confirm these findings in patients receiving immune-modulating agents.

## INTRODUCTION

Historically, the prognosis of patients with metastatic melanoma has been dismal, with a median survival of less than one year [1]. Standard chemotherapies such as dacarbazine, achieved objective responses in only 5–15% of patients, and failed to confer a survival benefit [2]. The recent advent of inhibitors of immune-checkpoints [cytotoxic T-lymphocyte antigen 4 (CTLA-4) and programmed cell death-1 (PD-1)], and of novel targeted therapies drastically improved survival expectations for advanced melanoma patients [3].

As different therapeutic options are becoming available for melanoma, there is an urgent need of predictive biomarkers that help to identify which treatment each patient is most likely to benefit from.

Thus far, a number of markers showed significant associations with the prognosis of patients receiving checkpoint inhibitors, although, in the absence of control treatment arms, none was formally proven to predict benefit from a given therapy [4, 5]. Recently, low baseline LDH-ratio, absence of visceral metastasis other than soft-tissue/lung, high relative lymphocyte count (RLC) and high relative eosinophil count (REC) were shown to be associated with improved survival of melanoma patients receiving the PD-1 blocker pembrolizumab [6].

In this retrospective study, we investigated if and how these parameters could inform on patients receiving another immuno-modulating agent, the anti-CTLA-4 ipilimumab. By comparing the outcomes of patients presenting or not the proposed favorable markers when treated with an anti-CTLA-4 or with chemotherapy, we identified baseline REC as a candidate predictive biomarker for benefit from ipilimumab.

## RESULTS

### Patient and treatments

A total of 244 patients were included in this study, and grouped according to the treatment received (Table 1). Cohort A included 116 patients (49 women and 67 men, with a median age of 57 years) who were treated with chemotherapy alone (*n* = 98) or combined with a target therapy (*n* = 18); at blood sampling, 78% of patients were receiving their first-line therapy. Cohort B included 128 patients (47 women and 81 men, with a median age of 60 years) who were treated with anti-CTLA-4 alone (*n* = 117) or combined with chemotherapy (*n* = 11); ipilimumab was a first-line therapy for 23% of patients. The two cohorts of patients were well balanced for patient and disease characteristics (Table 1), except for LDH (which was elevated in the majority of cohort B but not cohort A patients). Moreover, the four markers of interest (LDH-ratio, pattern of distant metastasis, RLC and REC) were observed at similar frequencies in the two cohorts of patients (Table 1).

**Table 1:** Patients and disease characteristics

	Cohort A	Cohort B	*p* value
**Age, years**			0.10
Median (IQR)	57 (48–67)	60.5 (53–71)	
**Gender, *n* (%)**			0.38
Women	49 (42)	47 (37)	
Men	67 (58)	81 (63)	
**Primary site, *n* (%)**			0.18
Cutaneous	90 (78)	100 (78)	
Mucosal	7 (6)	11 (9)	
Ocular	5 (4)	10 (8)	
Unknown	14 (12)	7 (5)	
**AJCC stage, *n* (%)**			0.75
3c + M1a + M1b	25 (22)	26 (20)	
M1c	88 (78)	101 (80)	
Not known	3	1	
**Serum LDH, *n* (%)**			0.02
< ULN	65 (61)	57 (45)	
≥ ULN	42 (39)	69 (55)	
Not available	9	2	
**Previous lines of therapy, *n* (%)**			< 0.0001
0	90 (78)	29 (23)	
≥ 1	26 (22)	99 (77)	
**LDH-ratio, *n* (%)**			0.84
≤ 2.5	96 (90)	112 (89)	
> 2.5	11 (10)	14 (11)	
**Pattern of metastasis, *n* (%)**			0.39
Lymph nodes, soft tissues, lung	33 (28)	43 (34)	
Other visceral	83 (72)	85 (66)	
**Relative lymphocyte count (RLC), *n* (%)**			0.41
< 17.5%	35 (30)	45 (35)	
≥ 17.5%	81 (70)	83 (65)	
**Relative eosinophil count (REC), *n* (%)**			0.41
< 1.5%	61 (53)	74 (58)	
≥ 1.5%	55 (47)	54 (42)	

Cohort A: 116 patients receiving chemotherapy; cohort B: 128 patients receiving anti-CTLA-4. *p* values are from Mann-Whitney test (age) or chi-square test (all other variables). Abbreviations: AJCC: American Joint Committee on Cancer; IQR: interquartile range; LDH: lactate dehydrogenase; n: number of patients; ULN: upper limit of normal.

Median follow-up was 25 and 11 months for patients of cohort A (*n* = 11) and B (*n* = 48), respectively, who were alive at the last follow-up, and 6.1 and 6.6 months, respectively, for the whole patient populations. Subsequent treatments included ipilimumab for four (3%) cohort A patients, and an anti-PD-1 for 0 and 27 (21%), respectively, cohort A and B patients.

### Baseline biomarkers and overall survival

First, we analyzed the associations of known prognostic factors (age, sex, stage and LDH) and of the recently proposed markers for pembrolizumab (LDH-ratio, pattern of distant metastasis, RLC and REC [6]) with the overall survival (OS) of patients receiving chemo-based therapy or anti-CTLA-4-based therapy. Four cohort A patients who subsequently received ipilimumab, were excluded from this analysis. Twenty-seven cohort B patients who later received pembrolizumab or nivolumab were censored at first anti-PD1 infusion. The median OS was 6.1 months for cohort A patients, and 8.0 months for cohort B patients.

**Figure 1:**
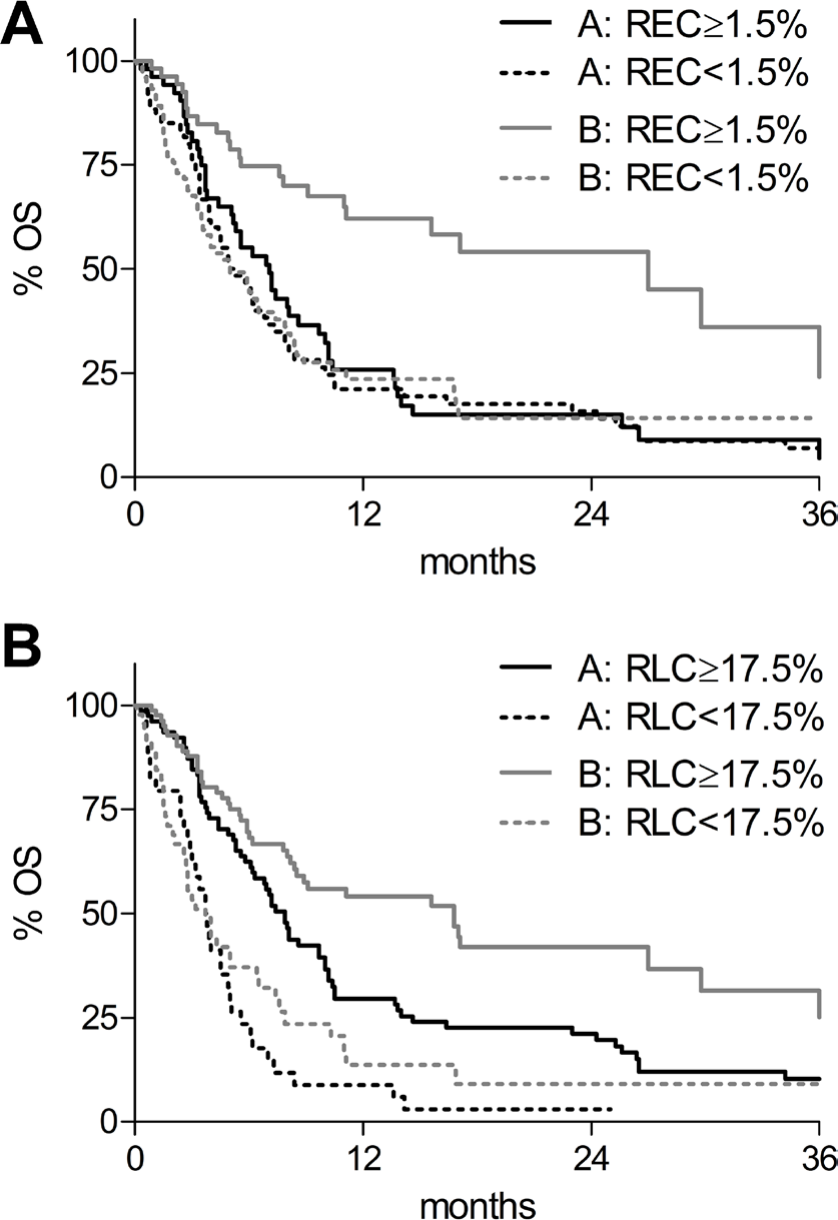
Kaplan-Meier overall survival (OS) curves according to biomarkers and therapies (**A**) OS according to baseline relative eosinophil count (REC). REC < 1.5%: dotted lines; REC ≥ 1.5%: solid lines. (**B**) OS according to baseline relative lymphocyte count (RLC). RLC < 17.5%: dotted lines; RLC ≥ 17.5%: solid lines. Black lines: chemotherapy-treated patients (cohort A); grey lines: anti-CTLA-4-treated patients (cohort B).

In univariate analysis (Supplementary Table 1), LDH-ratio ≤ 2.5, absence of visceral metastasis and RLC ≥ 17.5% were all significantly associated with improved survival in both cohorts of patients. Interestingly, REC ≥ 1.5% was associated with a favorable outcome for patients receiving anti-CTLA-4 (Supplementary Table 1 and Figure 1A, grey lines; *p* < 0.0001), but not with the prognosis of patients receiving chemotherapy (Supplementary Table 1 and Figure 1A, black lines; *p*= 0.43).

The median OS was 27.0 and 7.1 months, respectively, for patients with REC ≥1.5% treated with anti-CTLA-4 or with chemotherapy (continuous lines), and 5.0 months for patients with REC < 1.5% receiving both treatments (Figure 1A, dotted lines). Thus, patients with REC ≥ 1.5% seemed to derive much greater benefit from anti-CTLA-4 (HR = 0.41; 95% CI: 0.25–0.66; *p* = 0.0003; Figure 1A: continuous grey compared with black line), than patients with REC < 1.5% (HR = 0.95; 95% CI: 0.65–1.39; *p* = 0.80; Figure 1A: dotted grey compared with black line). Multivariate analysis identified a significant relationship of chosen treatment with baseline REC for outcome (*p* = 0.04, Table 2), confirming that patients with REC ≥ 1.5% had an improved survival compared to patients with REC < 1.5% only if they received anti-CTLA-4 treatment (HR = 0.56; 95% CI: 0.34–0.93, Table 2), Instead, no difference was observed for patients receiving chemotherapy (HR = 1.13; 95% CI: 0.74–1.74, Table 2). These data showed that REC ≥ 1.5% might be a predictive biomarker of response to ipilimumab.

**Table 2:** Multivariate cox proportional hazard models

	*n*	Overall survival		Progression free survival	
		HR (95% CI)	*p* value	HR (95% CI)	*p* value
**LDH-ratio**			< 0.0001		0.002
≥ 2.5	25	1.00 (Ref)		1.00 (Ref)	
< 2.5	208	0.28 (0.17–0.45)		0.49 (0.32–0.77)	
**Visceral metastases**			0.02		0.001
present	161	1.00 (Ref)		1.00 (Ref)	
absent	72	0.65 (0.44–0.94)		0.57 (0.41–0.79)	
**Relative lymphocyte count**			< 0.0001		0.002
< 17.5%	75	1.00 (Ref)		1.00 (Ref)	
≥ 17.5%	158	0.45 (0.32–0.63)		0.62 (0.46–0.84)	
**Treatment by REC**			0.04		0.009
chemo and REC < 1.5%	58	1.00 (Ref)		1.00 (Ref)	
chemo and REC ≥ 1.5%	49	1.13 (0.74–1.74)		1.38 (0.92–2.07)	
anti-CTLA-4 and REC < 1.5%	73	0.83 (0.57–1.22)		0.79 (0.55–1.13)	
anti-CTLA-4 and REC ≥ 1.5%	53	0.56 (0.34–0.93)		0.73 (0.48–1.10)	

Abbreviations: CI: confidence interval; HR: hazard ratio; LDH: lactate dehydrogenase; n: number of patients; REC: relative eosinophil count; Ref: reference.

On the other hand, baseline RLC was associated with patient’s prognosis in both groups of treatment. The median OS were 7.9 and 3.8 months, respectively, for cohort A patients with RLC ≥ 17.5% or lower (Supplementary Table 1 and Figure 1B, grey lines; *p* < 0.0001), and 16.8 and 3.7 months, respectively, for cohort B patients with RLC ≥ 17.5% or lower (Supplementary Table 1 and Figure 1B black lines; *p* < 0.0001). As compared to cohort A patients with RLC < 17.5%, multivariate analysis confirmed that patients with RLC ≥ 17.5% had a reduced risk of mortality if they were treated with anti-CTLA-4 (HR = 0.31; 95% CI: 0.19–0.51) but also if they received chemotherapy (HR = 0.52; 95% CI: 0.33–0.83).

### Baseline biomarkers and disease progression

The associations of the above described variables with the disease progression were then evaluated. One cohort A patient was excluded from this analysis, due to missing date of progression. The median progression free survival (PFS) was 1.9 months for cohort A patients, and 3.3 months for cohort B patients.

**Figure 2:**
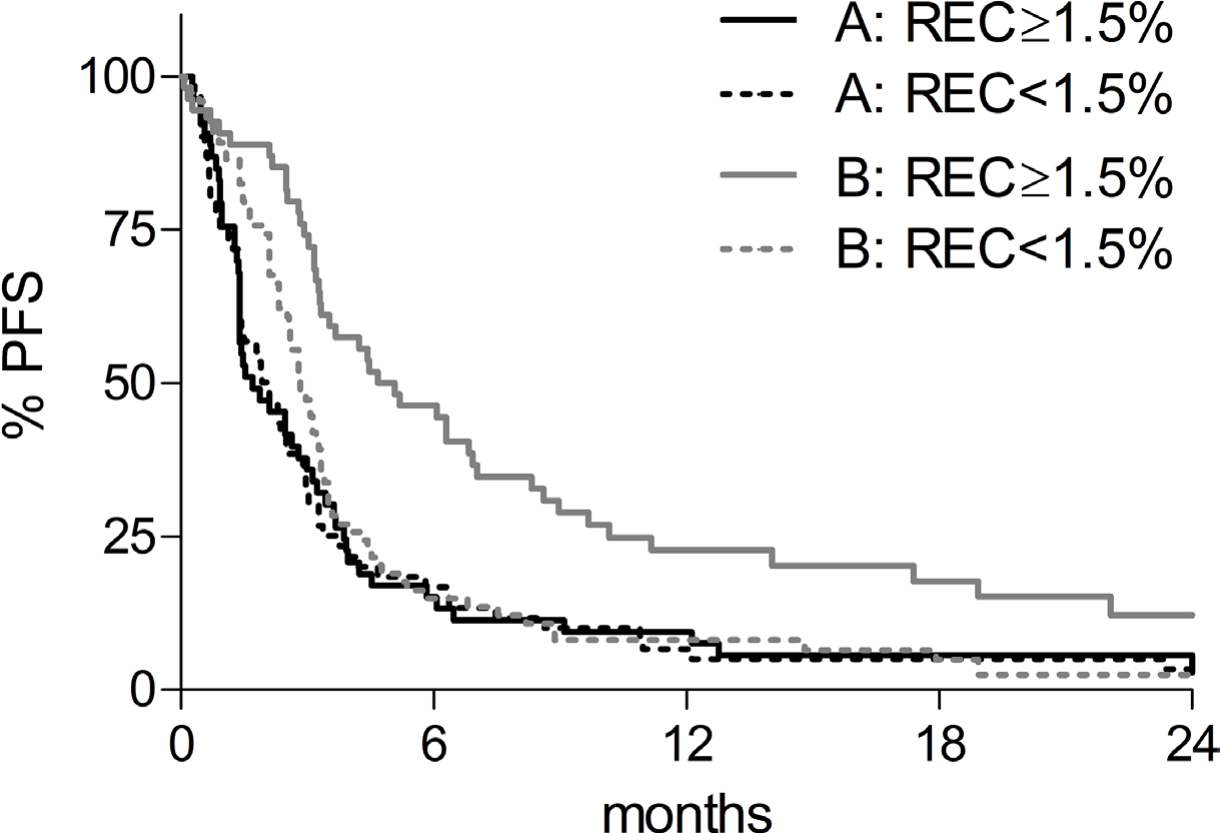
Kaplan-Meier progression free survival (PFS) curves according to baseline REC REC < 1.5%: dotted lines; REC ≥ 1.5%: solid lines. Black lines: chemotherapy-treated patients (cohort A); grey lines: anti-CTLA-4-treated patients (cohort B).

Similarly to what we observed for OS, univariate analysis (Supplementary Table 2) showed that LDH-ratio ≤ 2.5, absence of visceral metastasis and RLC ≥ 17.5% were all significantly associated with delayed progression in both cohorts of patients, while REC ≥ 1.5% was associated with a favorable outcome in patients receiving anti-CTLA-4 (*p* = 0.0003), but not in patients receiving chemotherapy (*p* = 0.89). As shown in Figure 2, the median PFS was 1.7 and 2.1 months for cohort A patients with REC ≥ 1.5% (black continuous line) or lower (black dotted line), and 4.9 and 2.8 months for cohort B patients with REC ≥ 1.5% (grey continuous line) or lower (grey dotted line), respectively.

Multivariate analysis confirmed a significant treatment-by-REC interaction also for disease progression (*p* = 0.009, Table 2), although in this case also patients with REC < 1.5% seemed to derive some benefit from anti-CTLA-4 (Figure 2, dotted lines). Patients with REC ≥ 1.5% tended to have a delayed disease progression compared to patients with REC < 1.5% if they were treated anti-CTLA-4 (HR = 0.73; 95% CI: 0.48–1.10, Table 2), but not if they received chemotherapy (HR = 1.38; 95% CI: 0.92–2.07, Table 2), although differences were not significant in multivariate analysis.

## DISCUSSION

In the present study, we analyzed the associations of four markers with the survival of patients treated with chemotherapy or anti-CTLA-4, and we identified baseline REC as a potential predictive biomarker for benefit from anti-CTLA-4 therapy. To our knowledge, this is the first study to report a formal statistical testing that allowed to discriminate between prognostic and predictive values of candidate biomarkers in this context.

Ipilimumab improved survival of advanced melanoma patients in randomized clinical trials, but response rates are generally low and side effects are frequent [7, 8]. We and others have previously shown that simple baseline counts of peripheral blood cells may reflect a general immune status more prone or refractory to mount anti-tumor responses upon CTLA-4 inhibition [9–14]. Although we do not know the biological reasons supporting the role of eosinophils in this scenario, we could speculate that inflammation has an important role in setting a favorable or unfavorable microenvironment. Tumor infiltrating lymphocytes (TILLs) as well as T regulatory cells (TREGs) or even myeloid cells, all known to influence the immunoediting balance, could produce/secrete cytokines attracting “specific” (a subset of Th17 could be involved in the process) or “generic” cells (such as eosinophils, neutrophils, macrophages) in order to influence specific or innate immunity [15].

The availability of novel therapeutic options for melanoma patients demands for biomarkers able to predict treatment benefit and assist clinical management decisions. A combined model consisting of pattern of distant metastasis, serum LDH and peripheral blood RLC and REC has been recently proposed for melanoma patients treated with the anti-PD-1 pembrolizumab [6]. While the absence of visceral metastasis and low serum LDH levels are known favorable prognostic factors for melanoma [1], RLC and REC are less characterized markers. Although a different cut-off was used, baseline RLC were found to be associated with OS of ipilimumab-treated patients in a previous study [11]. Here, we observed that RLC ≥ 17.5% was associated with a significantly reduced risk of mortality in patients receiving either chemotherapy or anti-CTLA-4. In contrast, REC ≥ 1.5% had no prognostic value for patients treated with chemotherapy, while halved the risk of mortality in patients receiving anti-CTLA-4. Multivariate testing confirmed an interaction between anti-CTLA-4 therapy and REC ≥ 1.5% for improved OS and PFS, indicating REC as a predictive biomarker for benefit from ipilimumab. Notably, patients with baseline REC ≥ 1.5% had a similar two-fold reduced risk of mortality when treated with pembrolizumab [6], suggesting that eosinophil might be a more general predictive marker for benefit from immune-checkpoint inhibitors. This hypothesis needs to be tested.

Associations of absolute baseline eosinophil counts with outcome of patients receiving ipilimumab have been documented [11]. Moreover, an early increase of circulating eosinophil during therapy was also associated with benefit from ipilimumab [12, 16], while another study showed a positive association with OS but not with clinical responses [17]. Whether eosinophils are directly involved in licensing or favoring anti-CTLA-4 induced responses, or they represent the epiphenomenon of still undetermined patient features, remains to be established, and the mechanisms underlying the beneficial effects of eosinophils need to be investigated.

This is a retrospective evaluation, which implies the difficulty for controlling potential confounding bias and demands validation in randomized trials. Major limitations include: 1) the diversity of the two cohorts of patients, and 2) the possible survival bias due to the disproportion of patients who received a subsequent anti-PD-1 therapy. Firstly, the majority (78%) of cohort A patients were receiving their first-line therapy, while only 23% of cohort B patients were treatment-naïve when they received anti-CTLA-4. Although the frequency of patients presenting with favorable baseline markers was comparable in the two cohorts, we cannot exclude an effect of previous treatments on our observations. Secondly, the two cohorts were largely non-overlapping in time. As a drawback, none of cohort A patients had access to anti-PD-1 therapy, whereas 21% of cohort B patients received this treatment after progression to the anti-CTLA-4. As overall survival could be confounded by subsequent treatment with these drugs [18–20], we censored these patients at their first anti-PD-1 infusion. Furthermore, REC retained their predictive value also for disease progression, which could not be affected by changes in patient standard of care.

In conclusion, this study provides evidences of the predictive impact of baseline REC for benefit from anti-CTLA-4 therapy, and calls for validation in randomized trials with ipilimumab and other immune-modulating agents.

## MATERIALS AND METHODS

### Patient selection

Main clinical information of melanoma patients was retrieved via three sources: 1) Tumor Registry of the European Institute of Oncology (IEO) included all patients admitted in the period 2000–2010. Use of the data included in the Tumor Registry was approved by the Institutional Review Board in March 2013. 2) IEO256 database included all melanoma patients who had a molecular test in IEO Pathology Unit in the period 2010–2014. 3) IEO255 database included all consecutive patients treated with ipilimumab at IEO. IEO255 and IEO256 studies were approved by Institutional Ethical Committee in July 2015, and patient informed consents were obtained. The follow-up was closed on 30th June 2016.

Main inclusion criteria were: diagnosis of advanced melanoma (unresectable Stage IIIc or Stage IV) [1], available pre-therapy blood test (performed 0–28 days before therapy initiation), and treatment with chemotherapy (either alone or combined with a target therapy for patients included in cohort A) or with an anti-CTLA-4 (either alone or combined with chemotherapy for patients included in cohort B). For patients who received multiple lines of therapy including both chemotherapy and anti-CTLA-4, only the first available blood test was considered, and patients were included in the appropriate study cohort. This study conforms to the Declaration of Helsinki and successive amendments.

### Statistical analysis

The four biomarkers of interest (LDH-ratio, pattern of distant metastasis, RLC and REC) were defined as described [6]. LDH-ratio was calculated dividing measured LDH value by the upper limit of normal. Patient and disease characteristics of the two cohorts were compared using Mann-Whitney and Chi-square tests, as appropriate. Progression free survival was calculated from first treatment to disease progression or death (event), or last follow-up (censored). Overall survival was calculated from first treatment to first anti-PD-1 infusion (censored) or death (event) or last follow-up (censored). Survival probabilities were estimated with the Kaplan-Meier method and compared using the Log-rank test. In order to evaluate the predictive effect of treatment by REC and RLC, multivariate Cox proportional hazard models adjusted for LDH-ratio and pattern of distant metastasis, were used to assess interaction between treatment effect (cohort factor) and biomarkers. Adjusted hazard ratios (HR) with 95% confidence intervals (CIs) were reported. All analyses were carried out with Prism (Graph Pad) and SAS software, version 9.2 (SAS Institute, Cary, NC). All reported tests were two-sided, and *p* < 0.05 was considered significant.

## SUPPLEMENTARY MATERIALS TABLES



## Abbreviations

CTLA-4: cytotoxic T-lymphocyte antigen 4
CI: confidence interval
HR: hazard ratio
LDH: lactate dehydrogenase
OS: overall survival
PD-1: programmed cell death-1
PFS: progression free survival
RLC: relative lymphocyte counts
REC: relative eosinophil counts
