# Supplementary material for: Baseline relative eosinophil count as a predictive biomarker for ipilimumab treatment in advanced melanoma
Source: Oncotarget. 2017 Aug 1;8(45):79809–15. doi: 10.18632/oncotarget.19748 (PMC5668095; doi:10.18632/oncotarget.19748)
Supplement: Supplementary file 1 [file oncotarget-08-79809-s001.pdf]

Baseline relative eosinophil count as a predictive biomarker for ipilimumab treatment in advanced melanoma

SUPPLEMENTARY MATERIALS

Supplementary Table 1: Univariate analysis for overall survival

	Cohort A		Cohort B	
	HR (95% CI)	*p* value	HR (95% CI)	*p* value
**Age**<br>≤ 60 vs > 60 years	1.21 (0.81–1.79)	0.36	0.75 (0.48–1.17)	0.20
**Gender**<br>Women vs Men	0.86 (0.58–1.27)	0.45	0.89 (0.56–1.41)	0.62
**AJCC stage**<br>3c + M1a + M1b vs M1c	0.61 (0.40–0.92)	0.02	0.52 (0.32–0.86)	0.01
**Serum LDH**<br>< ULN vs ≥ ULN	0.24 (0.14–0.42)	< 0.0001	0.59 (0.38–0.92)	0.02
**LDH-ratio**<br>≤ 2.5 vs > 2.5	0.00 (0.00–0.02)	< 0.0001	0.05 (0.02–0.16)	< 0.0001
**Visceral mets**<br>absent vs present	0.63 (0.42–0.94)	0.02	0.45 (0.29–0.71)	0.0005
**Relative lymphocyte count**<br>≥ 17.5% vs < 17.5%	0.29 (0.17–0.50)	< 0.0001	0.28 (0.17–0.48)	< 0.0001
**Relative eosinophil count**<br>≥ 1.5% vs < 1.5%	0.85 (0.57–1.27)	0.43	0.40 (0.26–0.63)	< 0.0001

Cohort A: patients receiving chemotherapy; cohort B: patients receiving anti-CTLA-4. *p* values and hazard ratios (HR) for mortality are from Log-rank test. Abbreviations: AJCC: American Joint Committee on Cancer; CI: confidence interval; LDH: lactate dehydrogenase; ULN: upper limit of normal.

Supplementary Table 2: Univariate analysis for progression free survival

	Cohort A		Cohort B	
	HR (95% CI)	*p* value	HR (95% CI)	*p* value
**Age**<br>≤ 60 vs > 60 years	1.16 (0.79–1.72)	0.45	0.96 (0.66–1.38)	0.82
**Gender**<br>Women vs Men	0.91 (0.62–1.34)	0.63	0.83 (0.57–1.21)	0.34
**AJCC stage**<br>3c + M1a + M1b vs M1c	0.65 (0.43–0.99)	0.04	0.42 (0.28–0.63)	< 0.0001
**Serum LDH**<br>< ULN vs ≥ ULN	0.32 (0.18–0.52)	< 0.0001	0.60 (0.42–0.88)	0.009
**LDH-ratio**<br>≤ 2.5 vs > 2.5	0.15 (0.05–0.42)	0.0003	0.17 (0.07–0.41)	< 0.0001
**Visceral mets**<br>absent vs present	0.67 (0.45–0.99)	0.04	0.27 (0.25–0.55)	< 0.0001
**Relative lymphocyte count**<br>≥ 17.5% vs < 17.5%	0.59 (0.38–0.94)	0.03	0.46 (0.30–0.71)	0.0004
**Relative eosinophil count**<br>≥ 1.5% vs < 1.5%	0.97 (0.67–1.43)	0.89	0.50 (0.34–0.73)	0.0003

Cohort A: patients receiving chemotherapy; cohort B: patients receiving anti-CTLA-4. *p* values and hazard ratios (HR) for disease progression are from Log-rank test. Abbreviations: AJCC: American Joint Committee on Cancer; CI: confidence interval; LDH: lactate dehydrogenase; ULN: upper limit of normal.
